# Joint segmentation of sternocleidomastoid and skeletal muscles in computed tomography images using a multiclass learning approach

**DOI:** 10.1007/s12194-024-00839-1

**Published:** 2024-09-06

**Authors:** Kosuke Ashino, Naoki Kamiya, Xiangrong Zhou, Hiroki Kato, Takeshi Hara, Hiroshi Fujita

**Affiliations:** 1 Graduate School of Information Science and Technology, Aichi Prefectural University, 1522-3 Ibaragabasama, Nagakute, Aichi 480-1198 Japan; 2 Faculty of Engineering, Gifu University, 1-1 Yanagido, Gifu, Gifu 501-1193 Japan; 3 Department of Radiology, Graduate School of Medicine, Gifu University, 1-1 Yanagido, Gifu, Gifu 501-1193 Japan; 4 Center for Healthcare Information Technology (C-HiT), Tokai National Higher Education and Research System, Nagoya, Aichi 464-8601 Japan

**Keywords:** Computed tomography images, Sternocleidomastoid muscle, Skeletal muscle, Segmentation

## Abstract

Deep-learning-based methods can improve robustness against individual variations in computed tomography (CT) images of the sternocleidomastoid muscle, which is a challenge when using conventional methods based on probabilistic atlases are used for automatic segmentation. Thus, this study proposes a novel multiclass learning approach for the joint segmentation of the sternocleidomastoid and skeletal muscles in CT images, and it employs a two-dimensional U-Net architecture. The proposed method concurrently learns and segmented segments the sternocleidomastoid muscle and the entire skeletal musculature. Consequently, three-dimensional segmentation results are generated for both muscle groups. Experiments conducted on a dataset of 30 body CT images demonstrated segmentation accuracies of 82.94% and 92.73% for the sternocleidomastoid muscle and entire skeletal muscle compartment, respectively. These results outperformed those of conventional methods, such as the single-region learning of a target muscle and multiclass learning of specific muscle pairs. Moreover, the multiclass learning paradigm facilitated a robust segmentation performance regardless of the input image range. This highlights the method’s potential for cases that present muscle atrophy or reduced muscle strength. The proposed method exhibits promising capabilities for the high-accuracy joint segmentation of the sternocleidomastoid and skeletal muscles and is effective in recognizing skeletal muscles, thus, it holds promise for integration into computer-aided diagnostic systems for comprehensive musculoskeletal analysis. These findings are expected to enhance medical image analysis techniques and their applications in clinical decision support systems.

## Introduction

Sarcopenia is characterized by an extreme age-related decline in skeletal muscle mass and strength [1]. In particular, it is a registered disease under the International Classification of Diseases (ICD). However, its unified diagnostic criteria remain open [2]. The Asian Working Group for Sarcopenia (AWGS) has proposed the use of appendicular skeletal muscle mass, grip strength, and gait speed as diagnostic criteria [3]. Furthermore, frailty (physical frailty) represents an intermediate state between independence and the requirement of nursing care [4]. Consequently, five items have been suggested as the diagnostic criteria for frailty: weight loss, exhaustion, low physical activity, slow gait speed, and weakness [5]. Both sarcopenia and frailty are associated with decreased skeletal muscle quantity and function; thus, skeletal muscle mass is a crucial indicator for care prevention. Skeletal muscle analysis using computed tomography (CT) images has been recognized as an effective method for diagnosing sarcopenia [6]. Hanaoka et al. proposed an analysis of the psoas major muscle using cross-sectional CT images at the third lumbar vertebra (L3) level [7]. Similarly, the skeletal muscles at level L3 [8] and the surface muscles of the entire body [9] have been segmented in several studies. In diagnostic support based on skeletal muscle segmentation and analysis, the site-specific muscles are important, as well as the entire skeletal musculature [10].

This study focuses on the sternocleidomastoid muscle, which is a powerful muscle that runs along the lateral neck [11]. The sternocleidomastoid muscle plays a crucial role in various movements of the head and neck, such as rotation of the head to the opposite side and flexion and extension of the neck. Electromyographic analysis of the sternocleidomastoid muscle can effectively differentiate amyotrophic lateral sclerosis [12] from cervical spondylotic myelopathy, which presents with similar initial symptoms [13]. The distribution of Hounsfield units (HU) for site-specific skeletal muscles, including the sternocleidomastoid muscle, overlaps with that of organs and other skeletal muscles in CT images. This overlap makes it difficult to distinguish between these regions using threshold-based segmentation techniques. Automatic segmentation of the sternocleidomastoid muscle has been attempted in body CT images using bone positional information at the anatomical attachment sites and a probabilistic atlas [14]. Thus, automatic segmentation of the sternocleidomastoid muscle has been researched for computer-aided diagnosis (CAD) [15]. However, the sternocleidomastoid muscle is not entirely visible in body CT images, which presents a challenge to landmark acquisition and alignment for atlas-based recognition methods. Consequently, the average recognition accuracy across 20 cases remains limited to 65.4%.

In contrast, the psoas major muscle, which is another specific skeletal muscle, is fully visible in body CT images and has a simpler shape than does the sternocleidomastoid muscle. This has enabled model-based recognition with an accuracy of 72.3% [16]. In recent years, deep learning approaches, particularly methods based on U-Net [17], have been successfully applied to muscle segmentation. Hashimoto et al. achieved segmentation of the psoas major muscle in low-dose CT images [18]. Furthermore, Castiglione et al. achieved skeletal muscle segmentation at the third lumbar vertebra with U-Net-based methods [8]. The potential of deep-learning-based automatic feature selection has been noted for skeletal muscle segmentation and could produce more accurate musculoskeletal analysis than conventional methods that manually select features [19]. Furthermore, U-Net-based segmentation models for 104 anatomical structures have been proposed [20]. Thus, deep learning-based methods for medical image segmentation have demonstrated their effectiveness not only for skeletal muscles but also in various other areas including organs and bones. Given this background, we conducted a study to compare between the conventional atlas-based method and a novel deep-learning-based approach for the three-dimensional (3D) automatic segmentation of the sternocleidomastoid muscle in body CT images.

The aim of this study is to achieve the joint segmentation of the sternocleidomastoid muscle and other skeletal muscles by applying deep learning techniques to the automatic segmentation of the sternocleidomastoid muscle. It is important not only to recognize the sternocleidomastoid muscle with higher accuracy than that achieved by conventional probability atlas-based recognition methods, but also to simultaneously enable comprehensive analysis of skeletal muscles. To accomplish this, we propose a joint segmentation method for the sternocleidomastoid muscle and other skeletal muscles using multiclass learning with a 2D U-Net [17] architecture. The aim is to segment the sternocleidomastoid muscle region. Unlike existing deep learning approaches that focus solely on the target muscle region, the proposed method simultaneously learns the sternocleidomastoid muscle and the complete skeletal musculature, and it facilitates the acquisition of 3D segmentation results for both the target sternocleidomastoid muscle and the entire skeletal muscle compartment. By incorporating knowledge from the surrounding muscle context, the proposed method enhances the segmentation performance for the sternocleidomastoid muscle region. Our method represents an advancement in applying U-Net architectures to skeletal muscle segmentation and marks a breakthrough in addressing the accuracy challenges that have confronted previous skeletal muscle segmentation methods. The proposed method simultaneously learns the sternocleidomastoid muscle and the entire skeletal musculature, and it facilitates the acquisition of 3D segmentation results for both the target sternocleidomastoid muscle and the entire skeletal muscle compartment. Therefore, the proposed method is expected to achieve site-specific skeletal muscle segmentation and comprehensive skeletal muscle analyses. The effectiveness of the proposed method was validated through a comparison with conventional methods.

## Materials and methods

### Image dataset

The image dataset comprised 30 non-contrast body CT images acquired at Gifu University Hospital. In this context, the 30 cases comprised images acquired from different patients, none of whom presented musculoskeletal disorders. The scanning device was a LightSpeed Ultra 16 (GE Healthcare). The spatial resolution was 0.625 $\times$ 0.625 $\times$ 0.625 [mm], and the image size was 512 $\times$ 512 $\times$ 802-1104 [voxels].

### Experimental environment

The experimental setup for this study was as follows. The computer was equipped with a Ryzen Threadripper PRO 5965WX CPU, NVIDIA RTX A6000 (48 GB $\times$ 3) GPUs, and 256 GB RAM, although the process could be executed on an NVIDIA Quadro RTX 5000 (16 GB$\times$1) GPU. The operating system was Ubuntu 20.04 LTS, and the deep learning library used was TensorFlow [21] 2.6.0+nv.

### Joint segmentation of sternocleidomastoid and skeletal muscles

**Fig. 1:**
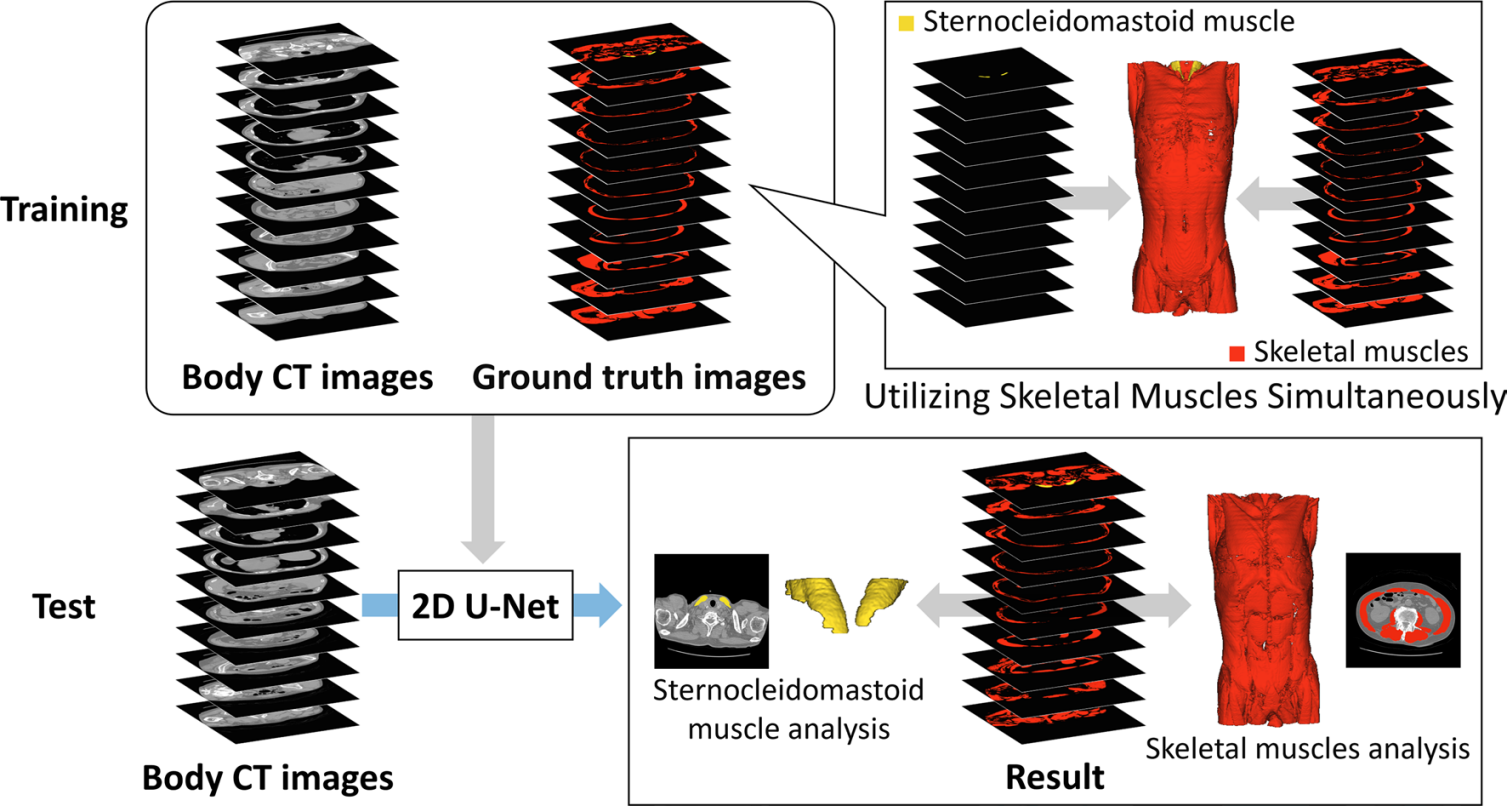
Segmentation of sternocleidomastoid muscle using multiclass learning with skeletal muscles (proposed method)

An overview of the proposed method is shown in Fig. 1. This study focused on the depiction of skeletal muscles in all cross-sectional slices within the scanning range of body CT images. Previously, Kawamoto et al. [22] proposed a method for recognizing skeletal muscles around the L3 cross-section by learning a site-specific muscle and the erector spinae muscle simultaneously. In contrast, our method focuses on skeletal muscles that are visible over a wider range. The main proposal of this work was to facilitate the automatic segmentation of the sternocleidomastoid muscle using a two-dimensional (2D) U-Net [17] through multiclass learning of sternocleidomastoid and skeletal muscles in the input body CT images. The U-Net architecture has demonstrated accuracies of 87.24% for COVID-19 lesion segmentation and 97.75% for lung segmentation in CT images [20]. These accuracies are within 1% of those for the successor network, the multi-transformer U-Net [23], which achieved accuracies of 88.18% and 98.01%, respectively. Moreover, U-Net was employed as the base architecture in recently proposed networks. Thus, U-Net is a promising network for benchmark segmentation-based methods. This study proposes a learning and segmentation method using 2D U-Net.

The process begins with inputting 3D body CT images and corresponding ground truth labels. The 2D U-Net is then trained on all axial slices extracted from these 3D volumes, and it learns to segment multiple muscle classes simultaneously. After it is trained, the U-Net processes each axial slice of a new 3D body CT image for segmentation. Finally, the individual 2D segmentation results are reconstructed to form a complete 3D segmentation of the sternocleidomastoid and other skeletal muscles. This method leverages the efficiency of 2D U-Net processing while effectively handling 3D volumetric data, resulting in a comprehensive 3D muscle segmentation. The 2D U-Net architecture consists of an encoder and a decoder. In this study, the encoder applies a process of two 3$\times$3 convolutions followed by 2$\times$2 max pooling for downsampling, repeated four times. Subsequently, two additional 3$\times$3 convolutions are applied. The decoder then employs a process of 2$\times$2 upsampling, concatenation with the corresponding encoder feature maps, and two 3$\times$3 convolutions, iterated four times. Batch normalization and rectified linear unit (ReLU) activation are applied after each 3$\times$3 convolution. Finally, a 1$\times$1 convolution followed by a softmax function is utilized. This architecture enables the segmentation of the sternocleidomastoid muscle and skeletal muscles.

The proposed method considered a three-class classification problem that distinguished between the sternocleidomastoid muscle, skeletal muscle, and background regions. Therefore, the loss function, same as in [21], and used respective weights of 0.5 and 1 for cross-entropy (CE) and Dice (loss = $0.5 \times CE + Dice$). And the learning parameters were set as follows: the number of epochs was 50, learning rate was $3 \times 10^{-4}$, batch size was four, and optimization function used was Adam [24]. In addition, data augmentation was applied to the training images, which is effective in the skeletal muscle segmentation of the lower and upper legs [25]. Specifically, augmentation techniques such as scaling, translation, rotation, shear transformation, and flipping were randomly applied to expand the training data size by a factor of eight.

### Experimental settings

In this study, the automatic segmentation of the sternocleidomastoid muscle was performed using a 2D U-Net based on multiclass learning with skeletal muscles. In such deep learning-based methods, segmentation through the learning of a single region of the target is the most fundamental approach. Therefore, in this study, we compare our method with the recognition results obtained from learning only the sternocleidomastoid muscle region as a baseline approach. Furthermore, as a deep learning-based method for the automatic recognition of specific skeletal muscles, a technique involving multiclass learning of the erector spinae muscle was previously proposed [22]. The multiclass learning of the target region and erector spinae muscles effectively segmented six regions: the trapezius, supraspinatus, rectus abdominis, obliquus abdominis, quadratus lumborum, and psoas major muscles. Therefore, this study performed segmentation through the multiclass learning of the sternocleidomastoid and erector spinae muscles and compared the results with our own method.

The learning and segmentation of only sternocleidomastoid muscle is a two-class classification problem. It distinguishes between the target sternocleidomastoid muscle and background regions. Consequently, the loss function employed a combination of the binary cross-entropy (BCE) and Dice loss, with respective weights of 0.5 and 1 (loss = $0.5 \times BCE + Dice$), and the sigmoid function was employed as the activation function as in a previous method [22]. However, the method uses multiclass learning for the sternocleidomastoid muscle and the erector spinae muscle results in a three-class classification problem, which is similar to the proposed method. Therefore, we use a loss function that employs CE and Dice, as described in Sect. 2.3 (loss = $0.5 \times CE + Dice$) and the softmax function is used as the activation function. All other network architecture and hyperparameters are set to the same as those of the proposed method described in Sect. 2.3.

### Ground truth and evaluation metrics

The 2D U-Net is a supervised learning method. Therefore, this study required ground truth images for 30 cases, the same number as the image dataset in Sect. 2.1. This step was required for both the target sternocleidomastoid and skeletal muscles used in the proposed method and the erector spinae muscles used for comparison with the conventional method [22]. The ground truth images are created by the first author (K. A.), a student majoring in computer science, using thresholding and the graph cut tool [26] implemented in PLUTO [27], a common platform for the computer-aided diagnosis of medical images. For overall skeletal muscle regions, the results of threshold processing were manually corrected. For specific skeletal muscle regions, manual marking was performed at intervals of several slices, followed by the application of the graph cut method. These results were then iteratively refined by manual correction and ultimately verified by the second author (N. K.), who holds a Ph.D. in medical science.

In this study, the dataset was divided into training and test sets on a per-patient basis, and threefold cross-validation was employed as the method for accuracy verification. Using the experimental environment described in Sect. 2.2, we conducted the training for three sets simultaneously, assigning each set to a different GPU. Experiments were conducted using the proposed method and the two learning approaches described in Sect. 2.4. The effectiveness of the proposed method was validated based on the accuracy of segmentation. The evaluation metrics for segmentation accuracy included the Dice (Dice=$2 \times |A \cap B|/|A| + |B|$), Recall (Recall=$|A \cap B|/|A|$), and Precision (Precision=$|A \cap B|/|B|$). Here, *A* represents the target region in the ground truth image, and *B* represents the target region in the segmentation result. Dice, Recall, and Precision were, respectively, used to evaluate the overlap ratio, under-segmentation rate, and over-segmentation rate between the ground truth image and the segmentation result.

## Results

### Segmentation results for sternocleidomastoid muscle

The segmentation results of the sternocleidomastoid muscle using different learning methods based on the mean and standard deviation of the Dice score, Recall, and Precision are listed in Table 1.

**Table 1:** Accuracy of the sternocleidomastoid muscle [%]

	SCM		
	- (Baseline)	+ESM [22]	+SKM (proposed)
Dice	0.00 ± 0.00	78.89±8.53	82.94±6.91
Recall	0.00±0.00	83.49±13.96	80.25±9.59
Precision	N/A	77.19±10.60	86.58±7.00

SCM: sternocleidomastoid muscle; ESM: erector spinae muscle; SKM: skeletal muscles

**Fig. 2:**
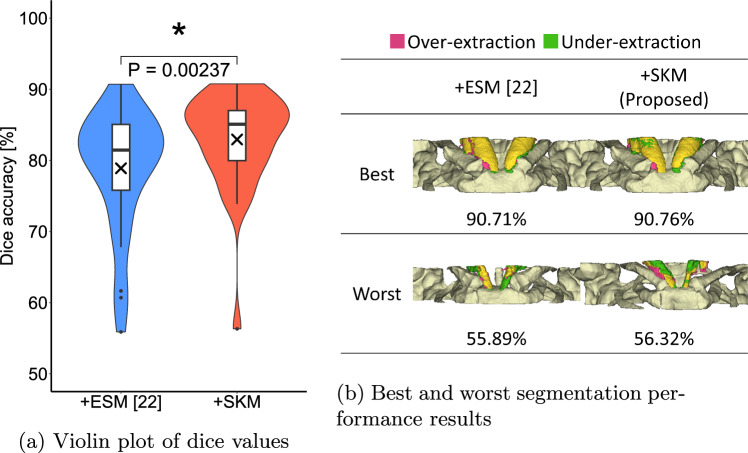
Dice of the sternocleidomastoid muscle: distribution and best/worst cases. ESM: erector spinae muscle; SKM: skeletal muscles

Although the sternocleidomastoid muscle could not be segmented when only the sternocleidomastoid muscle region was learned, segmentation was possible using the proposed method with multiclass learning considering the erector spinae muscles. Figure 2 illustrates the distribution of the Dice scores for the segmentation results of the sternocleidomastoid muscle using the proposed method and multiclass learning with the erector spinae muscles. Further, the segmentation results for the cases with the highest and lowest Dice scores are presented. Notably the segmentation accuracies of the proposed method exceeded 70% for a larger number of cases compared with multiclass learning with the erector spinae muscles, regardless of the best- or worst-case scenario. Furthermore, the proposed method achieves a 4.05% higher accuracy compared to the multiclass learning of the erector spinae muscle. Focusing on the 9.39% improvement in precision value from Table 1, this is due to the ability to prevent over-segmentation of the sternocleidomastoid muscle through joint segmentation of all other skeletal muscles, including the erector spinae. Moreover, the segmentation accuracy of the proposed deep learning-based method for 30 cases is 17.5% higher than the results of 20 cases using the conventional atlas-based method [14], which demonstrates the robustness and effectiveness of the proposed approach.

### Segmentation results for skeletal muscles

The proposed method, which employs the multiclass learning of the sternocleidomastoid and skeletal muscles, facilitated the simultaneous segmentation of the entire skeletal musculature and the sternocleidomastoid muscle. After the two segmented regions were integrated, the segmentation accuracy for the entire skeletal muscle compartment was 92.73% ± 5.14. In contrast, single-region learning for segmenting the entire skeletal muscle region yielded an accuracy of 87.18% ± 5.81, which was 5.55% lower than that of the proposed method. Previous study on automatic segmentation of the entire skeletal musculature [8] were limited to the cross-sectional area of the third lumbar vertebra (L3), achieving an accuracy of 93%. However, the proposed method achieved high-accuracy 3D segmentation of the entire skeletal muscle over a wider range than the L3 level.

## Discussion

### Comparison with segmentation methods restricting input image range

As shown in Sect. 3.1, in the segmentation task that used single-region learning, all voxels in the body CT images were classified as background region. Wakamatsu et al. demonstrated the effectiveness of the restricting the input slice range for the segmentation of the supraspinatus muscle in body CT images using U-Net [28]. The supraspinatus muscle was visible in only a limited range of CT slices in the body CT images. Consequently, it is considered that the imbalance between the target region and the background region in deep learning [29] led to unstable segmentation accuracy. In the image dataset, the maximum depiction range of the sternocleidomastoid muscle was only 9.64% relative to the scanning range of the torso. Therefore, we verified the effectiveness of the Wakamatsu method [28] in recognizing the sternocleidomastoid muscle using deep learning and compared it with our proposed method. A comparative experiment was conducted by restricting the input range of CT slices based on the anatomical features of the sternocleidomastoid muscle. The origin was the entire superior surface of the manubrium sterni, and the insertion was the superior surface of the medial one-third of the clavicle [11]. Specifically, the input range of CT slices for the 2D U-Net was manually restricted from the top of the scanning range in the image dataset to the range wherein the inferior border of the first rib was depicted. Consequently, the size of the restricted image dataset and ground truth images was 512 $\times$ 512 $\times$ 69-119 [voxels].

Table 2 presents the segmentation accuracies for the sternocleidomastoid muscle when the input range of CT slices was restricted to 2D U-Net. Note that the mean Dice score for the single-region learning of the sternocleidomastoid muscle region improved from 0.00% to 73.92% ($Wilcoxon, p < 0.05$) owing to the restriction of the input range of CT slices. This result demonstrated the effectiveness of restricting the input range of CT slices based on the bone position information for single-region learning and segmentation of the sternocleidomastoid muscle. Further, the segmentation accuracy improved by 2.78% ($Wilcoxon, p < 0.05$) for multiclass learning with the erector spinae muscles. Therefore, restricting the input range of CT slices was effective for both single-region learning of the sternocleidomastoid muscle and multiclass learning of the erector spinae muscles. However, the mean Dice score for the sternocleidomastoid muscle in the proposed multiclass learning with skeletal muscles was 81.92% when the input range of the CT slices was restricted. This was 1.02% lower than that in the case without an input range restriction ($Wilcoxon, p > 0.05$). From this, it can be inferred that the information from the range of CT slices where the sternocleidomastoid muscle is not depicted contributed to stabilizing the multiclass learning in our proposed method. However, the proposed multiclass learning approach for the segmentation of the sternocleidomastoid muscle achieved the highest segmentation accuracy regardless of the input range of CT slices. Moreover, the proposed method exhibited a higher accuracy without the restriction of the input range of CT slices based on the estimated depiction range of the sternocleidomastoid muscle. Thus, this segmentation method did not require an input range restriction based on bone position information.

**Table 2:** Accuracy of the sternocleidomastoid muscle in cropped images [%]

	SCM		
	- (Baseline)	+ESM [22]	+SKM (proposed)
Dice	73.92 ± 13.72	81.67 ± 8.82	81.92 ± 7.67
Recall	68.56 ± 19.14	81.28 ± 12.24	79.12 ± 11.92
Precision	85.45 ± 10.28	83.69 ± 10.27	86.45 ± 7.89

SCM: sternocleidomastoid muscle; ESM: erector spinae muscle; SKM: skeletal muscles

### Relationship between depiction range, sternocleidomastoid muscle volume, and segmentation accuracy

Section 4.1 demonstrated that restricting the input range of the CT slices improved the segmentation accuracy of the sternocleidomastoid muscle for single-region learning and the multiclass learning of the erector spinae muscles using a 2D U-Net. This is because restricting the input range of the CT slices increased the depiction range of the sternocleidomastoid muscle compared to the scanning range of the image dataset. Therefore, the relationship between the size of the sternocleidomastoid muscle and segmentation accuracy was examined.

**Fig. 3:**
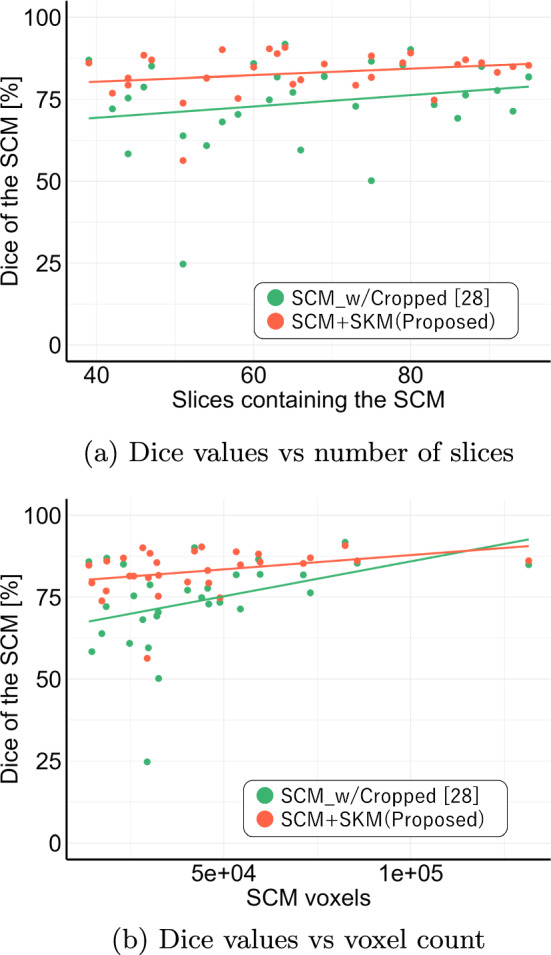
Obtained Dice and size of the sternocleidomastoid muscle using proposed method. Red: proposed method; green: single-task learning with input range restriction

Figure 3 depicts the distribution of segmentation accuracies for the proposed method (in red) and the baseline method of single-region learning with input range restriction (in green) based on the number of slices depicting the sternocleidomastoid muscle and its volume in the image dataset. Note that although both segmentation methods exhibit a similar trend in the relationship between the segmentation accuracy and the number of slices depicting the sternocleidomastoid muscle, the proposed method improved the segmentation accuracy regardless of the depiction range. Furthermore, the proposed method outperformed the baseline method, which restricted the input range of CT slices based on the bone position information [28], particularly for cases with smaller sizes of the depicted sternocleidomastoid muscle. This result implies that the proposed method is a robust segmentation approach regardless of the scanning range or size of the sternocleidomastoid muscle and is expected to be effective even in cases with muscle atrophy or reduced muscle strength.

In the proposed method, the case with the lowest segmentation accuracy for the sternocleidomastoid muscle, as indicated by the violin plot in Fig. 2, was an outlier. However, this case did not correspond to the minimum or maximum depicted range or volume of the sternocleidomastoid muscle (Fig. 3). Instead, it exhibited a particularly large depiction of the mandible, which is uncommon in image datasets. Thus, cases, wherein the mandible is depicted with a different posture during scanning compared to the rest of the dataset, remain a challenge for the proposed method and will be addressed in future work. In this study, the training labels were generated by two non-clinician authors, because the targets are normal structures in muscle regions with relatively well-defined boundaries on CT images. In addition, the volumes of target regions are considerably larger than the pixel-level manual annotation errors of their boundaries, so we consider the impact on performance is expected to be minimal. However, it is considered that more precise annotation by clinicians may be required when expanding the training dataset to include abnormal structures in order to enhance the robustness of the proposed method.

## Conclusion

In previous research, the 3D automatic recognition of the sternocleidomastoid muscle has been limited to recognition with the use of an established atlas, and no deep learning approaches have been explored. Additionally, although deep learning has been applied to the automatic recognition of skeletal muscles as a whole, it has been limited to the L3 cross-section, and 3D recognition remains open. This study proposed a multiclass learning approach for the joint segmentation of the sternocleidomastoid and skeletal muscles using a 2D U-Net architecture. The proposed method achieved segmentation accuracies of 82.94% for the sternocleidomastoid muscle and 92.73% for the entire skeletal muscle compartment. The proposed method demonstrated not only higher accuracy than the conventional atlas-based method for automatic recognition of the sternocleidomastoid muscle [14], but also surpassed the performance of deep learning-based methods that have shown effectiveness in recognizing other specific skeletal muscles [22, 28]. Furthermore, the proposed method was able to realize high-accuracy segmentation regardless of the input range of slices, owing to the fact that skeletal muscles are depicted in all cross-sectional slices of CT images. These results suggest that the proposed method is effective for recognizing skeletal muscles, leading to a comprehensive analysis of the skeletal muscular system in the trunk region. However, cases in which the mandible was depicted, exhibited lower segmentation accuracy compared to other cases, albeit with improvements over conventional methods. Future work will focus on enhancing robustness by not only applying U-Net-based successor networks, but also expanding the training dataset by including open data, and normalizing posture variations.

## Data Availability

The data that support the findings of this study are available from the authors but restrictions apply to the availability of these data, which were used under license from the IRB of Aichi Prefectural University and Gifu University for the current study, and so are not publicly available. Data are, however, available from the authors upon reasonable request and with permission from the IRB of Aichi Prefectural University and Gifu University.
